# Corrigendum to Integrating hypertension and diabetes management in primary health care settings: HEARTS as a tool

**DOI:** 10.26633/RPSP.2022.206

**Published:** 2022-11-30

The *Pan American Journal of Public Health* draws readers’ attention to an error in the following article, pointed out by the authors.

In page 1, affiliation of *William H. Herman should read number 5* Department of Epidemiology, University of Michigan, Ann Arbor, United States of America

